# RETRACTION: A Meta‐Analysis of Topical Ketorolac's Effect on Surgical Site Wound Healing Post‐Cataract Surgery

**DOI:** 10.1111/iwj.70578

**Published:** 2025-04-18

## Abstract

**RETRACTION:** YouR. and HanY. , “A Meta‐Analysis of Topical Ketorolac's Effect on Surgical Site Wound Healing Post‐Cataract Surgery,” International Wound Journal 21, no. 1 (2024): e14661, 10.1111/iwj.14661.38272819 PMC10789915

The above article, published online on 15 January 2024, in Wiley Online Library (http://onlinelibrary.wiley.com/), has been retracted by agreement between the journal Editor in Chief, Professor Keith Harding; and John Wiley & Sons Ltd. Following an investigation by the publisher, all parties have concluded that this article was accepted solely on the basis of a compromised peer review process. The editors have therefore decided to retract the article. The authors did not respond to our notice regarding the retraction.

**RETRACTION:** R. You and Y. Han , “A Meta‐Analysis of Topical Ketorolac's Effect on Surgical Site Wound Healing Post‐Cataract Surgery,” International Wound Journal 21, no. 1 (2024): e14661, 10.1111/iwj.14661.38272819 PMC10789915

The above article, published online on 15 January 2024, in Wiley Online Library (http://onlinelibrary.wiley.com/), has been retracted by agreement between the journal Editor in Chief, Professor Keith Harding; and John Wiley & Sons Ltd. Following an investigation by the publisher, all parties have concluded that this article was accepted solely on the basis of a compromised peer review process. The editors have therefore decided to retract the article. The authors did not respond to our notice regarding the retraction.

